# Protein inhibitor of retinal membrane guanylyl cyclase suppresses cGMP synthesis in photoreceptors

**DOI:** 10.1016/j.jbc.2025.110718

**Published:** 2025-09-15

**Authors:** Igor V. Peshenko, Elena V. Olshevskaya, Alexander M. Dizhoor

**Affiliations:** 1 Pennsylvania College of Optometry, Drexel University, Elkins Park, Pennsylvania, USA; 2 Department of Neurobiology and Anatomy, Drexel University, Philadelphia, Pennsylvania, USA

**Keywords:** guanylate cyclase (guanylyl cyclase), cyclic GMP (cGMP), photoreceptor, retina, retinal degeneration, vision, calcium binding proteins, RetGC, GCAP, signal transduction, phototransduction

## Abstract

Retinal membrane guanylyl cyclase (RetGC) regulated by RetGC activating proteins (GCAPs) imparts light sensitivity to rods and cones by producing cyclic GMP (cGMP) to open cGMP-gated channels in the photoreceptor outer segments. However, excessive cGMP synthesis by deregulated RetGC:GCAP complex provokes cone-rod degeneration and causes congenital blindness. We developed the first to date specific protein inhibitor of retinal guanylyl cyclase (PIGCY) capable of reducing RetGC activity in photoreceptor outer segment. PIGCY was constructed by modifying GCAP1 to eliminate its ability to activate RetGC while increasing its affinity for RetGC. PIGCY uncouples RetGC1:GCAP1 complex *in vitro*, reducing V_max_ and increasing K_mGTP_ of RetGC activity. PIGCY inhibits both basal and GCAP-stimulated RetGC activity from wildtype, *GCAP1*^*−/−*^, *GCAP2*^*−/−*^, and *GCAPs*^*−/−*^ mouse retinas homogenates and in isolated rod outer segments. When expressed in mouse rods, PIGCY accumulates in outer segments and decelerates endogenous GCAP-stimulated RetGC activity in *PIGCY*^*Tg*^ retina.

Retinal guanylyl cyclase (RetGC) in rod and cone outer segments produces second messenger of phototransduction, cyclic GMP (cGMP), to open cyclic nucleotide-gated channels (CNGCs). cGMP becomes hydrolyzed by light-activated phosphodiesterase 6 (PDE6) to close CNGC after illumination and generate hyperpolarization of the photoreceptor membrane as the first step in visual pathway (reviewed in (1, 2, 3, 4, 5)). There are two RetGC isozymes in photoreceptors—RetGC1 *(GUCY2D*), the main cyclase isozyme in rods and cones, and an ancillary isozyme, RetGC2 (*GUCY2F*), present in rods (3, 4, 6, 7, 8, 9). Ca^2+^/Mg^2+^ binding proteins, GCAPs, accelerate RetGC activity after illumination, when CNGC become closed and the influx of Ca^2+^ through the channels stops, thus converting GCAPs into a Mg^2+^-liganded state, and decelerate RetGC in the dark, when Ca^2+^ influx through CNGC resumes and Mg^2+^ in GCAPs becomes replaced by Ca^2+^ (4, 10). This negative Ca^2+^ feedback prevents uncontrolled increase of cGMP concentrations and restricts opening excessive number of CNGC channels in the dark. Various dominant mutations in *GUCY2D* and *GUCA1A*, the respective genes coding for RetGC1 and GCAP1 in a human genome, cause photoreceptor dystrophies (4, 11, 12, 13, 14, 15) by deregulating cGMP synthesis in the dark and therefore abnormally increasing Ca^2+^ influx through CNGC in the outer segment (16, 17, 18). Several approaches designed to prevent the deregulated RetGC activity in the dark have been tested in animal models of *GUCY2D-* and *GUCA1A*-linked retinopathies, including gene editing to eliminate the mutant allele (19), suppressing translation of the mutant allele gene product by siRNA (20), and acceleration of cGMP hydrolysis in the affected photoreceptors (21). The use of specific inhibitors of RetGC activity in the outer segment could be considered as yet another potential strategy for treating *GUCY2D* and *GUCA1A* retinopathies, but such inhibitors have never been identified to date. Here, we explored a possibility of creating of such RetGC-specific inhibitor named protein inhibitor of retinal guanylyl cyclase (PIGCY) for the potential use in gene therapy of retinal degenerations caused by overproduction of cGMP in the outer segment. We demonstrate that expression of PIGCY in living photoreceptors in a mouse model reduces the level of cGMP synthesis by RetGC.

## Results

### GCAP1 converted into PIGCY binds RetGC but does not activate it

**Figure 1:**
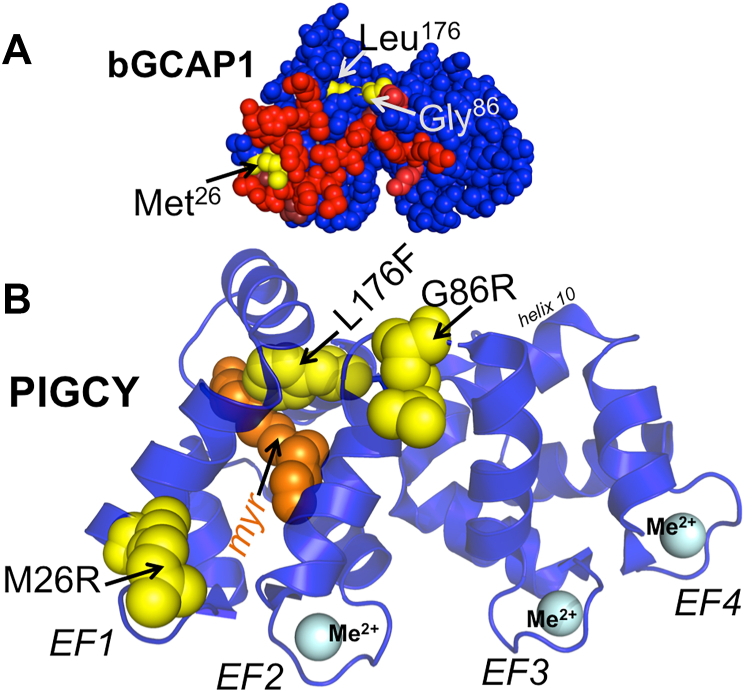
**Construction of PIGCY by mutagenesis of GCAP1.***A,* diagram of the residues for mutagenesis converting bovine GCAP1 into PIGCY (*yellow*); GCAP1 residues required for its binding to RetGC1 (ref. 22) are shown in *red*. *B,* location of the substituted residues (*yellow, filled spheres*) in a ribbon diagram of PIGCY tertiary structure. Arg^26^ in EF-hand 1 entry helix is located in the middle of the RetGC1 binding interface, substituting Met that is not required for the binding (ref. 22). Arg^86^ substitutes the “hinge” Gly between the EF-hand 1/EF-hand 2 and EF-hand 3/EF-hand 4 semi-globules (27). Phe^176^ substitutes Leu connecting the exiting helix of EF-hand 4 with the myristoyl (Myr, *orange*) moiety buried inside the EF-hand1/EF-hand 2 semi-globule *via* “calcium-myristoyl tug” involving helix 10; EF1 through EF4–Ca^2+^/Mg^2+^ (Me^2+^, *cyan*) binding loops of the corresponding EF-hand domains. The three-dimensional GCAP1 diagram is based on Stephen *et al.*, 2007, crystal structure (26). GCAP, RetGC activating protein; PIGCY, protein inhibitor of retinal guanylyl cyclase.

Two types of substitutions were introduced in GCAP1 to generate PIGCY (Fig. 1): (i) M26R, substitution in a nonmetal binding EF-hand 1, a part of the RetGC1 binding interface on GCAP1 (22, 23), was used to disable activation of RetGC by competing with GCAP1 and GCAP2 (24, 25); (ii) two other substitutions, G86R in a hinge region between the two semi-globules of GCAP1 (26, 27) and L176F in the alpha helix 11 connecting EF-hand 1 with EF-hand 4 *via* ‘calcium-myristoyl tug’ (28), were used to increase the M26R GCAP1 affinity for RetGC1.

**Figure 2:**
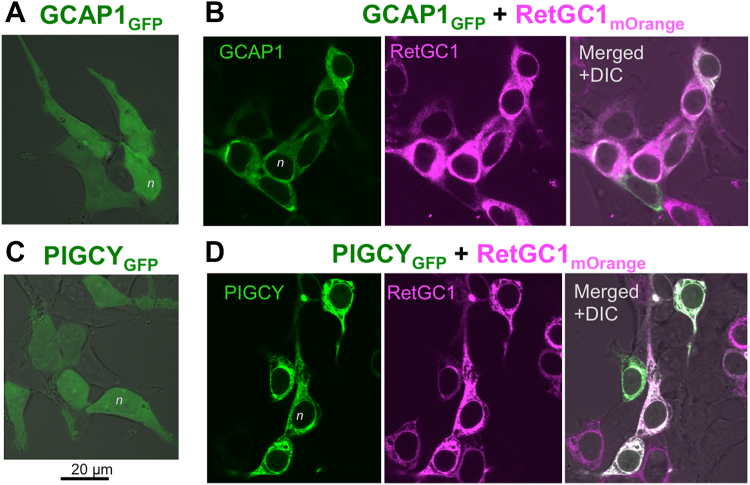
**PIGCY retains the ability to bind RetGC1 in HEK293 cells.***A–D,* GCAP1-GFP (*A*, *B*) and PIGCY-GFP (*C*, *D*) were expressed in HEK293 cells in the absence (*A*, *C*) or in the presence (*B*, *D*) of mOrange-RetGC1. Both GCAP1 and PIGCY display diffuse localization in the cytoplasm and the nuclei (*n*) in the absence of RetGC1, but when co-expressed with RetGC1 produce well-defined membrane colocalization pattern; in *B* and *D*, mOrange fluorescence was assigned *magenta color.* Fluorescence is superimposed on differential interference contrast (DIC) in *A* and *C* and the rightmost panels in (*B*) and *D*. The assay originally described in (31) was conducted with modifications described in (22, 24); see Experimental Procedures for additional details. GCAP, RetGC activating protein; PIGCY, protein inhibitor of retinal guanylyl cyclase; RetGC, retinal membrane guanylyl cyclase.

The ability of the resultant PIGCY variant, M26R/G86R/L176F, to bind RetGC1 was tested using a cell-based assay (Fig. 2) (22, 24), in which PIGCY tagged with GFP at the Cterminus was co-expressed with mOrange-tagged RetGC1. GCAPs affinity for RetGC lies in a micromolar range (9, 24, 29), and its complex with RetGC1 even in the outer segment membranes is rather labile, prone to dissociation upon washing the membranes (6, 30). Most critically, GCAP1 binding to RetGC1 cannot be assessed using co-immunoprecipitation because RetGC1:GCAP1 complex is completely inactivated and destroyed by detergents. In contrast, the cell-based assay can test the ability of GCAP1 to associate with RetGC1 by their colocalization in HEK293 cells (31). HEK293 cells do not possess mechanisms required for RetGC1 transport to the plasma membrane (32), therefore fluorescently tagged RetGC1 expressed in HEK293 cells accumulates predominantly in the membranes of the endoplasmic reticulum (ER). In contrast, GCAP1, as a part of the soluble 50-kDa GCAP1-GFP construct, spreads through the cytoplasm and karyoplasm of the cell, likely by simple diffusion, because nuclear pores do not commonly present barriers for diffusion of the proteins of that size. However, the tagged GCAP1 associates with the ER membranes containing RetGC1 when expressed using a low cDNA dosage relative to that of ReGC1 for cotransfection. This cell-based assay was previously correlated with the apparent affinity of GCAP1 and its mutants for RetGC1 in activation assay *in vitro* (22, 24). Anchoring of GCAP to the ER membranes becomes completely disrupted when mutations strongly reduce GCAP1 apparent affinity for RetGC1 in the activation assay *in vitro* (22, 24).

**Figure 3:**
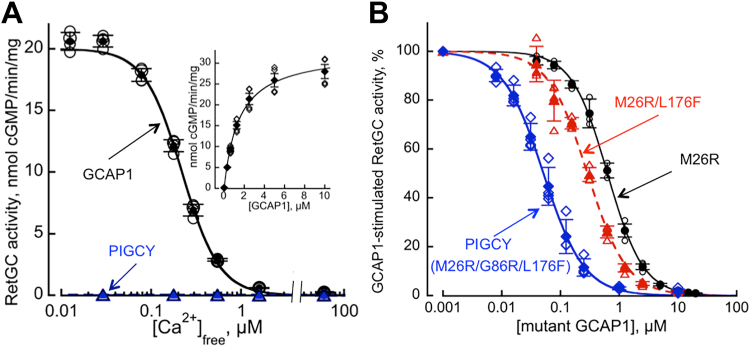
**PIGCY binding to RetGC1 without activating the cyclase suppresses RetGC1 activation by GCAP1.***A,* calcium-sensitivity of recombinant RetGC1 in the presence of 14 μM GCAP1 (○,•) or 14 μM PIGCY (,) assayed at the indicated [Ca^2+^ ]_free_ concentrations in the presence of 0.9 mM free Mg^2+^; *open symbols*: individual replicates; *filled symbols*: mean ± SD error bars. The data were fitted assuming a sigmoidal function, *A*_*max*_*/(1+([Ca]/[Ca]half)*^*h*^)*,* where *A*_*max*_ is the maximal cyclase activity, *[Ca]*is concentrations of free Ca^2+^ in the assay, and *h* is the Hill coefficient; *A*_*max*_ in the presence of wildtype GCAP1 and PIGCY in this experiment was 20 and 0 nmol cGMP/min/mg. Inset: a typical dose-dependence of RetGC1 activation by Mg^2+^GCAP1 in the absence of Ca^2+^ (◇,◆), a hyperbolic Michaelis fit. *B,* dose-dependent inhibition of the activated GCAP1/RetGC1 complex by GCAP1 mutants. The activity of recombinant RetGC1 in HEK293 membranes reconstituted with 1 μM GCAP1 was assayed at the indicated concentrations of M26R (○,•), M26R/L176F (,), and PIGCY (M26R/L176F/G86R) (,) in the presence of 2 mM EGTA and 10 mM MgCl_2_. The data were fitted assuming a sigmoidal function *A*_*%*_ = *100/(1+([GCAP1mut]/IC*_*50*_*)*^*h*^*)*, where *A*_*%*_ is a percentage of the normalized activity stimulated by 1 μM GCAP1 in each experiment, *[GCAP1mut]* is concentration of GCAP1 mutant, *h* is the Hill coefficient, and IC_50_ is the GCAP1 mutant concentration causing 50% inhibition (0.64 ± 0.09 μM, 0.3 ± 0.014, and 0.052 ± 0.001 μM, respectively; one-way ANOVA/Tukey test *p* < 0.001); independent measurements—*open symbols*, mean ± SD error bars—*filled symbols*. GCAP, RetGC activating protein; PIGCY, protein inhibitor of retinal guanylyl cyclase; RetGC, retinal membrane guanylyl cyclase.

Similar to wildtype GCAP1, GFP-tagged PIGCY in this cell-based assay was uniformly distributed throughout the cytoplasm and the nucleus of HEK cells when expressed alone but acquired a well-defined membrane-associated pattern due to colocalization with RetGC1 when the two proteins were co-expressed. However, unlike GCAP1, despite the ability of PIGCY to associate with RetGC1 in living HEK293 cells, purified recombinant PIGCY was completely unable to stimulate recombinant RetGC1 expressed in HEK293 cells *in vitro* (Fig. 3), even in the conditions being optimal for its activation by wildtype GCAP1 (low Ca^2+^/high Mg^2+^).

### PIGCY uncouples RetGC:GCAP complex *in vitro*

**Figure 4:**
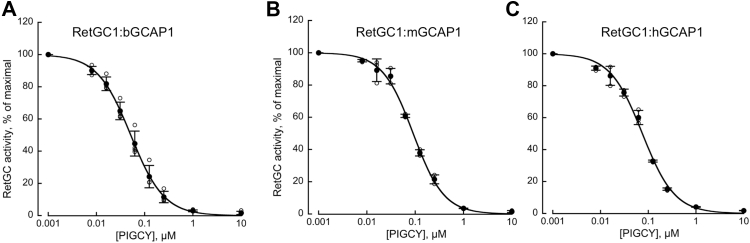
**PIGCY suppresses RetGC1 activation by different orthologs of GCAP1.***A–C*, inhibition by PIGCY of RetGC1 preactivated by 1 μM bovine (*A*), mouse (*B*) or human (*C*) recombinant GCAP1 in the presence of 2 mM EGTA and 10 mM MgCl_2_; the assays were performed as described in Fig. 3*B* legend, IC_50_ was 0.051; 0.091 and 0.075 μM; ○, data from independent measurements; •, mean ± SD error bars. The data were fitted assuming a sigmoidal function *A*_*%*_ = *100/(1+([PIGCY]/IC*_*50*_*)*^*h*^*)*, where *A*_*%*_ is a percentage of the maximal activity in each experiment, *h* is the Hill coefficient, and IC_50_ is the PIGCY concentration causing 50% inhibition. GCAP, RetGC activating protein; PIGCY, protein inhibitor of retinal guanylyl cyclase; RetGC, retinal membrane guanylyl cyclase.

As a result of its ability to bind RetGC1 without activating it, PIGCY competition with GCAP1 suppressed GCAP1-dependent stimulation of the cyclase activity *in vitro* (Fig. 3*B*). In a quasi-linear range of RetGC1 dose-dependence of activation on Mg^2+^GCAP1 (Fig. 3*A*, *inset*), this inhibition was detectable at low, less than 1:20, PIGCY:GCAP1 molar ratio. The IC_50_ for RetGC1:GCAP1 complex inhibition was ∼10-fold lower in the final M26R/G86R/L176F variant of PIGCY than the original M26R GCAP1 and 6-fold lower than M26R/G86R GCAP1 (Fig. 3*B*). Whereas Mg^2+^GCAP1 activates recombinant human RetGC1 with EC_50_ ∼1.5 μM (Fig. 3*A*, inset) (29), PIGCY was able to uncouple the RetGC1:GCAP1 complex containing bovine, mouse, or human GCAP1 at 17- to 30-fold lower concentrations (IC_50_ ∼50–90 nM) (Fig. 4, *A*–*C*).

**Figure 5:**
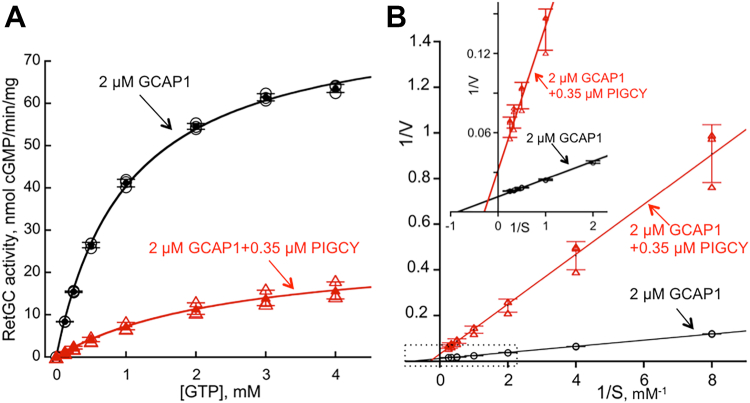
**PIGCY reduces V_max_ and increases K_m GTP_ of RetGC1 activated by GCAP1.***A,* the Michaelis plot of recombinant human RetGC1 activity in HEK293 cell membranes reconstituted with 2 μM wildtype GCAP1 at 0 mM Ca^2+^/10 mM MgCl_2_ in the absence (○, three independent measurements; •, mean ± SD) or in the presence of 0.35 μM PIGCY (, three independent measurements; , mean ± SD). *B*, Lineweaver*–*Burk plot of the data from panel A illustrating the change in V_max_ and K_mGTP_ parameters; *inset* – the expanded part (the *dashed box*) of the plot for 1/S = −1 to 2 mM^-1^ GTP^-1^. GCAP, RetGC activating protein; PIGCY, protein inhibitor of retinal guanylyl cyclase; RetGC, retinal membrane guanylyl cyclase.

PIGCY effect on RetGC1 activity was opposite from GCAP1. As GCAPs activate RetGC by increasing its V_max_ and reducing K_mGTP_ (6, 9), PIGCY counteracted the GCAP-dependent stimulation of the enzyme by reducing its V_max_ more than 3-fold (Student *t* test *p* = 0.00063) and increasing K_mGTP_ more than 2.5-fold (*p* < 0.0001) (Fig. 5, *A* and *B*).

**Figure 6:**
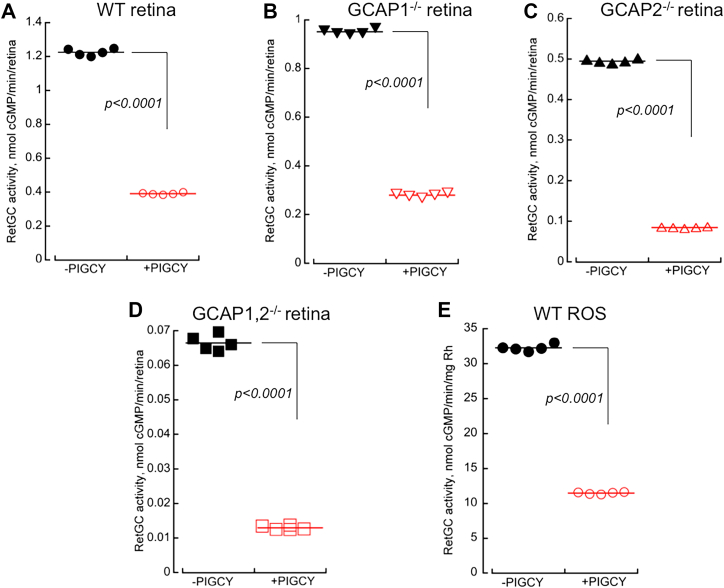
**Recombinant PIGCY inhibits native GCAP-stimulated and basal RetGC activity from a mouse retina.***A–E*, the endogenous RetGC activity at 0 Ca/0.9 mM Mg^2+^_free_ in crude retinal homogenates from wildtype (*A*, ,•,), *GCAP1*^*−/−*^ (*B*, ▼,), *GCAP2*^*−/−*^ (*C*, ▲,), and *GCAPs*^*−/−*^ double-knockout (*D*, ▪, ) retinas or isolated wildtype rod outer segment fraction (*E*, ,•,) assayed in the absence (*filled symbols*) or in the presence (*open symbols*) of 3 μM PIGCY; data points are independent measurements in multiple aliquots obtained for each retinal preparation; the *p* values are from Student unpaired *t* test assuming unequal variance. GCAP, RetGC activating protein; PIGCY, protein inhibitor of retinal guanylyl cyclase; RetGC, retinal membrane guanylyl cyclase; ROS, rod outer segment.

Purified recombinant PIGCY effectively uncoupled not only recombinant RetGC1:GCAP1 complex but also native RetGC:GCAP complexes preformed in the retina. When tested in whole-retinal homogenates of wildtype, *GCAP1*^*−/−*^, and *GCAP2*^*−/−*^ retinas, PIGCY inhibited the activities of the RetGC complexes in mouse retinas regulated by both endogenous GCAP1 and GCAP2 (Fig. 6, *A*–*C*). In addition to the activities of the endogenous RetGC stimulated by GCAPs, PIGCY added in both GCAP1- and GCAP2-deficient (*GCAPs*^*−/−*^) retina samples reduced the basal activity of RetGC (Fig. 6*D*). In rod outer segments (ROS) fraction isolated from mouse retinas using density gradient centrifugation, adding PIGCY reduced the total RetGC activity, from 32 to 11 nmol cGMP/min/mg rhodopsin (Fig. 6*E*).

### PIGCY expressed in rod outer segments reduces endogenous RetGC activity in transgenic mouse retinas

**Figure 7:**
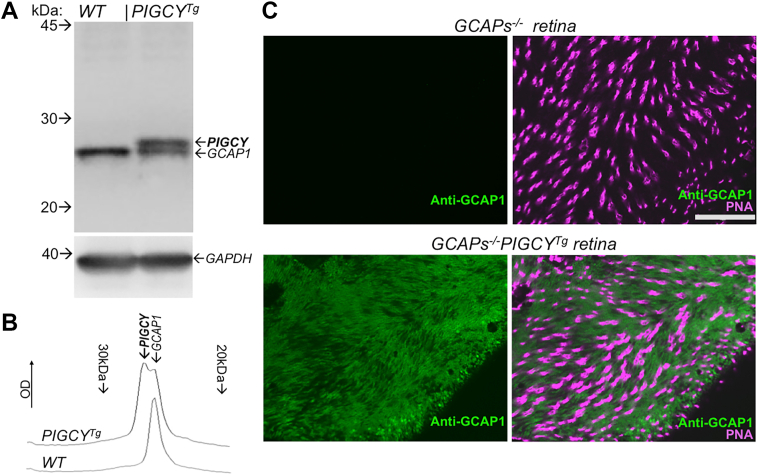
**Transgenic expression of PIGCY in mouse rods.***A,* immunoblotting of mouse retinas from wildtype and *PIGCY*^*Tg*^ littermates probed by anti-GCAP1 antibody (*top*); note the doublet band in *PIGCY*^*Tg*^ due to the presence of a lower-mobility product encoded by the transgene; *bottom panel*–GAPDH load control. *B,* PIGCY is expressed at a similar to the endogenous GCAP1 level; optical density profile of the images in *PIGCY*^*Tg*^ (*upper* trace) and wildtype (*lower* trace) retinal immunoblot probed by anti-GCAP1 antibody. *C*, flat-mount view of mosaic of PIGCY expression in 1.5-months-old *GCAPs*^*−/−*^*PIGCY*^*Tg*^ mouse retinas probed with anti-GCAP1 antibody (*green*) and PNA (peanut agglutinin, *magenta*) counterstaining cones; bar–60 microns. *GCAPs*^*−/−*^ retina used as a control is shown in the *top panel*; images for the two genotypes were taken in same experiment using the identical settings for image acquisition. GCAP, RetGC activating protein; PIGCY, protein inhibitor of retinal guanylyl cyclase.

**Figure 8:**
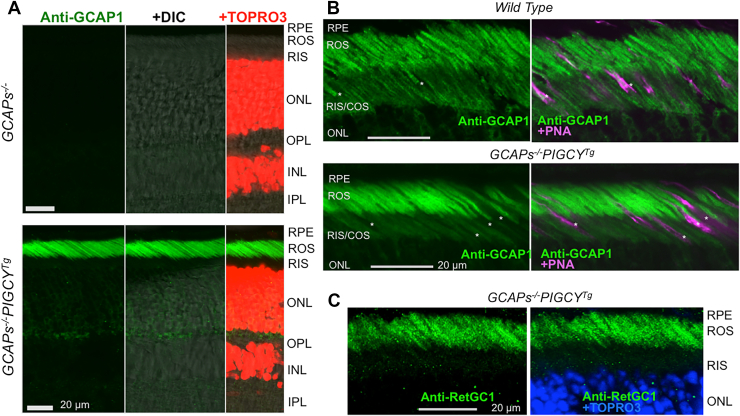
**PIGCY is delivered to rod outer segments.***A,* immunofluorescence in *GCAPs*^*−/−*^ and *GCAPs*^*−/−*^*PIGCY*^*Tg*^ retinas probed with anti-GCAP1 antibody; anti-GCAP1 fluorescence (*left panels*) is also shown superimposed on DIC image (*middle panels*) and additionally on TO-PRO-3 fluorescence (*right panels*); bar–20 μm. *B,* Anti-GCAP1 immunofluorescence (*green*) in wildtype (*top*) and *GCAPs*^*−/−*^*PIGCY*^*Tg*^ (*bottom*) shown either alone (*left panels*) or superimposed on fluorescence of cone sheaths counterstained with peanut agglutinin (PNA, *magenta*) in the *right panels*; note the presence of PIGCY in *GCAPs*^*−/−*^*PIGCY*^*Tg*^ rods but not cones (*asterisks*), in contrast to wildtype. *C*, Anti-RetGC1 fluorescence in *GCAPs*^*−/−*^*PIGCY*^*Tg*^; superimposed on TO-PRO-3 (*pseudo-blue*) in the *right panel*. RPE, retinal pigment epithelium; ROS, rod outer segments; RIS, rod inner segments; COS, cone outer segments; ONL, outer nuclear layer; OPL, outer plexiform layer; INL, inner nuclear layer; IPL, inner plexiform layer; GCAP, RetGC activating protein; PIGCY, protein inhibitor of retinal guanylyl cyclase.

The results observed with the purified recombinant PIGCY suggested that PIGCY would likely be able to inhibit the native RetGC:GCAP complexes when present in the outer segments *in vivo*, thus prompting us to generate a transgenic mouse model that expresses PIGCY using rod opsin promoter (*PIGCY*^*Tg*^) in order to directly test that possibility. PIGCY expressed in *PIGCY*^*Tg*^ retinas was detectable on Western immunoblot when probed by polyclonal anti-GCAP1 antibody (Fig. 7*A*). PIGCY migrates slightly slower than endogenous GCAP1 in SDS-PAGE electrophoresis, resulting in appearance of an additional protein band on the Western blot. Qualitative assessment based on the relative intensities of the upper PIGCY band *versus* the lower endogenous GCAP1 band in *PIGCY*^*Tg*^ (Fig. 7, *A* and *B*), indicated that PIGCY was likely expressed at a similar to the endogenous GCAP1 level in *PIGCY*^*Tg*^ rods. The same anti-GCAP1 antibody was also utilized for addressing the cellular localization of PIGCY, although that obviously required using instead of the wildtype as a genetic background the GCAP-deficient genotype (*GCAPs*^−/−^), lacking the endogenous GCAP1. The anti-GCAP1 antibody used to probe the *GCAPs*^*−/−*^*PIGCY*^*Tg*^ retina revealed not uncommon for transgenic protein expression mosaicism, cell-to-cell variation of PIGCY expression between *PIGCY*^*Tg*^ rods. However, this immunostaining also confirmed that the vast majority of rods in the transgenic retinas express PIGCY (Fig. 7*C*). Cellular distribution of PIGCY relative to the endogenous GCAP1 in *PIGCY*^*Tg*^ was not feasible to determine in wildtype background because anti-GCAP1 antibody does not discriminate between the two proteins. Therefore, the cellular localization of PIGCY was also assessed in *GCAPs*^*−/−*^*PIGCY*^*Tg*^ retinas (Fig. 8, *A* and *B*). Once the use of *GCAPs*^−/−^ background eliminated immunofluorescence of the endogenous GCAP1 normally present in wildtype rods and cones (Fig. 8, *A, B*), only anti-GCAP1 immunoreactivity of PIGCY remained detectable in *GCAPs*^*−/−*^*PIGCY*^*Tg*^. The immunostaining confirmed that PIGCY was expressed in rods but not cones and that it was delivered in ROS (Fig. 8, *A* and *B*), where RetGC1 was also present (Fig. 8*C*).

**Figure 9:**
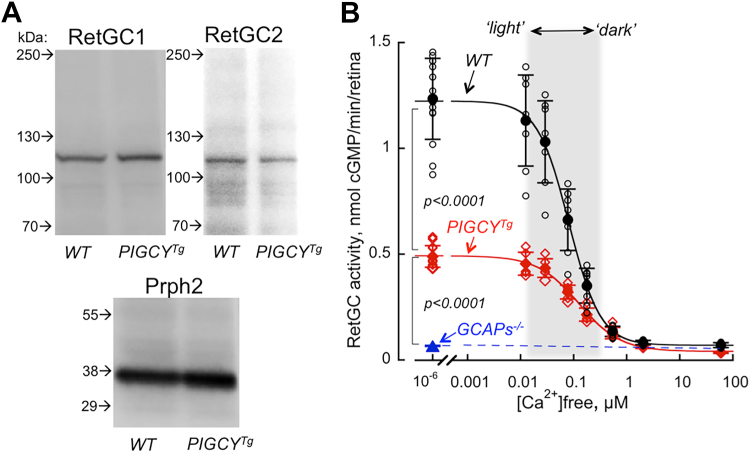
**Transgenically expressed PIGCY does not eliminate Ca^2+^ feedback but reduces the activity of endogenous RetGC in mouse retinas.***A,* Western immunoblot of wildtype and *PIGCY*^*Tg*^ retinas probed with anti-RetGC1 and anti-RetGC2 antibody; the peripherin-2 antibody (Prph2) was used as the loading control for the photoreceptor membrane-specific proteins of the retina. *B,* Ca^2+^-sensitive RetGC activity assayed in the presence of physiological (34) 0.9 mM free Mg^2+^ in wildtype (○,•) and *PIGCY*^*Tg*^ (,) retinas; data from independent measurements (*open symbols*) were averaged (*filled symbols*, ± SD error bars) and fitted assuming a sigmoidal Hill function; *shaded area–*the approximate range of free Ca^2+^ concentrations in mouse rod outer segments between light-adapted and dark-adapted states (16, 34); (,): Ca^2+^-insensitive (9) RetGC activity in *GCAPs*^*−/−*^ retinas for; *p* values are from Student *t* test. PIGCY, protein inhibitor of retinal guanylyl cyclase; RetGC, retinal membrane guanylyl cyclase.

PIGCY expression did not noticeably reduce the levels of expression of RetGC1 and RetGC2 (Fig. 9*A*) However, the rate of cGMP synthesis (mean ± SD) in *PIGCY*^*Tg*^ retinas when measured at low Ca^2+^ in the presence of 0.9 mM [Mg^2+^]_free_ (physiological conditions supporting stimulation of RetGC by GCAP1, ref. (33, 34)) was strongly reduced, to 0.49 ± 0.05 nmol cGMP/min/retina (n = 5), from 1.23 ± 0.2 nmol/min/retina in wildtype (n = 8, Student *t* test *p* < 0.0001) (Fig. 9*B*). However, cGMP synthesis in *PIGCY*^*Tg*^ retinas still remained Ca^2+^-sensitive and well above the 0.07 ± 0.02 nmol cGMP/min/retina in *GCAPs*^*−/−*^ (n = 5, *p* < 0.0001) (Fig. 9*B*).

### PIGCY does not provoke retinal degeneration

**Figure 10:**
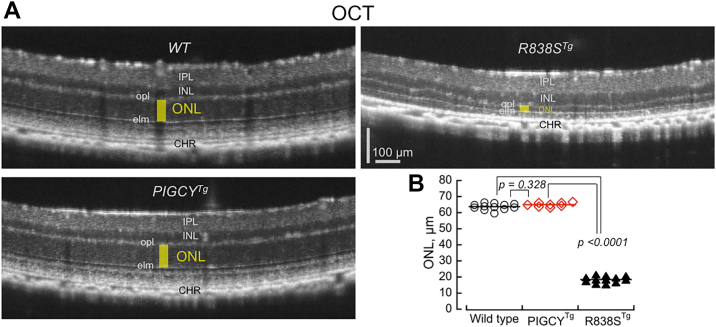
**PIGCY expression in rods does not provoke retinal degeneration.***A,* representative OCT B-scans at 3 months of age in wildtype, *PIGCY*^*Tg*^, and *R838S*^*Tg*^ mice (the latter is used for comparison as a degenerating retina example); the *yellow box* highlights the outer nuclear layer (ONL) thickness between outer plexifiorm (opl) and external limiting membrane (elm) reflective layers; other marked reflective layers are as follows: IPL—inner plexiform, INL—inner nuclear layer, CHR—choroid. *B,* quantification of the ONL thickness in wildtype (○) and PIGCY () *versus* R838S^Tg^ (▲) (mean ± SD, n): 63 ± 2, μm, 12; 65 ± 1.2 μm, 8; and 19 ± 2 μm, 12; *p* values: one-way ANOVA/Tukey test. OCT, optical coherence tomography.

Integrity of the photoreceptor layer in *PIGCY*^*Tg*^ retinas was evaluated *in vivo* by optical coherence tomography by measuring the thickness of the outer nuclear layer (ONL), which is composed of photoreceptor nuclei (Fig. 10, *A* and *B*). In a stark contrast to a mouse model undergoing photoreceptor degeneration caused by transgenic expression of a human R838S RetGC1 mutant (*R838S*^*Tg*^, ref. (35)), there was no detectable difference between *PIGCY*^*Tg*^ and wildtype retinas after 3 months of age—the ONL thickness remained unaffected by PIGCY expression (*p* > 0.3).

## Discussion

Two main potential approaches to prevent or reduce retinal degeneration caused by dominant mutations in *GUCY2D* and *GICA1A* leading to deregulation of cGMP synthesis in photoreceptors in the dark are being actively explored at present. The first is based on the idea of elimination of both the mutant and the wildtype alleles of *GUCY2D* using gene editing while expressing a normal allele modified to become insensitive to the CRISPR/Cas9 treatment (19). The second is based on counteracting accelerated cGMP synthesis in the dark by accelerating cGMP hydrolysis in the dark using ectopic expression of PDE5. Unlike light-dependent PDE6, PDE5 does not require activation by rhodopsin to rescue mouse photoreceptors harboring dominant mutant of RetGC1 or GCAP1 (21).

We reason that reduction of the RetGC1:GCAP1 complex activity should also be explored as one of the potential approaches to halt or decelerate retinal degenerations associated with the autosomal dominant *GUCA1A* and/or *GUCY2D* cone and rod dystrophies caused by deregulation of cGMP synthesis. Finding the inhibitors capable of selectively suppressing cGMP synthesis in photoreceptors *in vivo*, however, presents major challenge. Whereas RetGC activity can be suppressed *in vitro* by synthetic GTP analog guanosine tetrakisphosphate and some organic cations, such as benzamidine and octylamine (36), these inhibitors are nonselective for RetGC and are hardly suitable for using as therapeutic agents *in vivo*. Inhibitors suppressing guanylyl cyclase activity indirectly, such as inhibitors of NO synthase or inorganic pyrophosphatase, could not be used for such a purpose either, because NO regulates only soluble guanylyl cyclase (37, 38) but not membrane receptor guanylyl cyclases, such as RetGC, whereas pyrophosphatase inhibitor imidodiphosphate not only requires very high concentrations for inhibiting RetGC *in vitro* (36, 39) but would also affect a wide range metabolic pathways throughout the tissues and organs. For those reasons, the use of a specific protein inhibitor expressed in the affected photoreceptors using gene therapy application seems like a more reasonable approach, provided that such a protein or a peptide is able to bind RetGC with sufficiently high affinity and specificity to modulate the cyclase activity in the outer segments.

To date, two proteins were shown to be able to decrease RetGC activity. A presence of calcium-binding protein named GCIP was reported in photoreceptors of some lower vertebrates, fish and frog (40, 41). Recombinant frog GCIP produced in insect cells was shown to reduce RetGC activity in mammalian ROS membrane preparations *in vitro*. However, this inhibitory effect of GCIP was only demonstrated at Ca^2+^ concentration by far exceeding its normal levels in mammalian photoreceptors in the dark (40). It still remains unclear whether or not GCIP plays a physiological role *in vivo*, and it was even concluded that GCIP does not regulate RetGC in mammalian photoreceptors, lacking its ortholog (42).

Another protein, retinal degeneration-3 (RD3) (4, 32), acts as a very potent and highly selective inhibitor of RetGC activity in mammalian photoreceptors (43, 44). It also plays a critical role in RetGC delivery from the inner segment to the outer segment (32, 45, 46). However, RD3 localization is restricted to the inner segment, where it is needed for preventing aberrant activation of RetGC by GCAP before the cyclase reaches its proper destination in the outer segment, because the lack of that inhibition is detrimental for the photoreceptors (47, 48, 49, 50). Overexpression of RD3 slows degeneration of mouse photoreceptors harboring a human R838S RetGC1 (50), one of the *GUCY2D* mutations that cause adCORD (12, 51). The attenuating effect of RD3 in that case is based on its natural function—suppression of the aberrant activation of the mutant RetGC1:GCAP complex in the inner segment (43, 47, 48, 49). However, overexpression of RD3 only partially improves the survival of photoreceptors harboring R838S RetGC1 (50), because RD3 cannot eliminate the main “trigger” causing the cell death—deregulation of cGMP production in the outer segment (12, 18, 35). Binding of GCAP by RetGC when the latter enters outer segment causes displacement of RD3 from the cyclase, thus prohibiting RD3 access to the outer segment and forcing it to compartmentalize in the inner segment (46, 47, 48). As a result, the mutated RetGC:GCAP complex in the outer segment largely remains inaccessible by RD3.

In the present study, we designed the RetGC-specific inhibitor PIGCY by converting the natural RetGC-activating protein, GCAP1, into an artificial permanent inhibitory form competing with the normal GCAP1 for binding to RetGC1. Two important steps were involved in the process of creating PIGCY. First, PIGCY retains the main natural property of GCAP1—its unique ability to bind RetGC in the outer segment. In order to further increase PIGCY affinity for the cyclase, we utilized substitutions in GCAP1 associated with human *GUCA1A* retinopathies, such as G86R (27) and L176F (13). The L176F substitution in alpha helix 10 connecting the exiting helix of EF-hand 4 with the N-myristoyl residue embedded in the first semi-globule of GCAP1 (Fig. 1) increases GCAP1 affinity for RetGC1 *via* a “calcium-myristoyl tag” mechanism (28, 52). Second substitution, G86R, in the “hinge” between the two semi-globules of GCAP1 locks GCAP1 in its “RetGC activator” conformation while simultaneously increasing its affinity for RetGC1 (27). The higher affinity for the cyclase caused by these two mutations underlies the origin of *GUCA1A*-linked diseases because it contributes to shifting Ca^2+^-sensitivity of the cGMP synthesis outside the normal physiological range (13, 27, 28). The both mutations cause insufficient suppression of RetGC1:GCAP1 complex capable to create abnormal accumulation of cGMP in dark-adapted photoreceptors, when cGMP hydrolysis needs to be decelerated. To design PIGCY, we utilized the ability of the G86R and L176F substitutions to increase GCAP1 affinity for RetGC while preventing activation of the RetGC that these mutations would otherwise cause.

The key residue for converting GCAP1 into PIGCY was Met^26^ in EF-hand 1 (Fig. 1). Mapping the interface for RetGC1 binding on GCAP1 (22) identified Met^26^ as the residue located in the middle of the interface and surrounded by residues essential for RetGC1 binding. We previously demonstrated that the M26R substitution completely disables GCAP1 ability to activate RetGC without compromising its ability to bind the cyclase in HEK293 cells. The cell-based assay employed in these studies was originally developed to overcome the obstacle for evaluating the occurrence of binding to RetGC1 by other methods, because detergents required extraction and co-immunoprecipitation of RetGC:GCAP complexes from the membrane instantly inactivate and destroy these complexes. The evidence that the cell-based method adequately reflects the ability of GCAP1 to bind RetGC1 are three-fold: (i) GCAP1 expressed at low levels changes its localization from ubiquitously diffuse to ER-associated when RetGC1 is present in the ER membranes (31); (ii) this colocalization is independent from and does not require cGMP production by RetGC1 expressed in HEK293 cells (53); and (iii) numerous previously tested disease-related and artificial mutations in either RetGC1 or GCAP1 that can strongly reduce GCAP1 apparent affinity for RetGC1 based on the dose-dependence of RetGC1 activation by GCAP1 *in vitro* also prevent their colocalization in HEK23 cells, whereas multiple mutations that did not affect the apparent affinity of the GCAP1 for RetGC1 complex in the RetGC activation assay all demonstrate such colocalization (22, 24, 53, 54, 55). An exception from this pattern is the M26R GCAP1 mutant, which clearly showed colocalization but lacked the ability to activate RetGC1 *in vitro* (22, 24). Based on that, we concluded that M26R GCAP1 could act as a competing inhibitor for the normal GCAP1:RetGC1 complex, which was directly verified by the RetGC1 activity assays *in vitro* (22, 24, 25, 53). For that reason, M26R GCAP1 was chosen as the backbone further modified by the G86R and L1276F substitutions in order to make the M26R GCAP1 mutant a more effective inhibitor and turn it into PIGCY. The triple combination, M26R/G86R/L176F, which did not prevent the ability of the modified GCAP1 to colocalize with RetGC1 in a cell-based assay (Fig. 2), dramatically reduced the IC_50_ for inhibition of the RetGC1:GCAP1 complex activity, from micromolar to nanomolar range (Fig. 3). It is important to emphasize that despite this highly increased apparent affinity for the cyclase in the inhibition test, PIGCY, just like the original M26R GCAP1, completely lacked the ability to activate RetGC1 at both low and high free Ca^2+^ concentrations.

PIGCY reduces RetGC activity and effectively counteracts activation of the cyclase by both GCAP1 and GCAP2 *via* negative Ca^2+^ feedback *in vitro* (Fig. 6). Consequently, expression of PIGCY in transgenic rods was sufficient to substantially reduce cGMP production in the *PIGCY*^*Tg*^ photoreceptors (Fig. 9). This opens a possibility of testing PIGCY as a prospective inhibitor of RetGC *in vivo* to offset severe degeneration of photoreceptors caused by deregulated cGMP production. Therefore, it is also important that PIGCY expression *per se* does not provoke retinal degeneration, such as one caused by elevation of cGMP synthesis (16, 17, 35) (Fig. 10, *A* and *B*). This was not unexpected, because PIGCY in *PIGCY*^*Tg*^ retinas is expressed at the levels that apparently are not drastically different from the endogenous GCAP1. GCAP1 is a rather minor protein in rod outer segments, nearly 1000-fold less abundant than rhodopsin (9). In previous studies, overexpressed GCAP1 did not provoke rod death, unless it was affected by retinopathy-causing mutations (16, 17, 56). A vast majority of *PIGCY*^*Tg*^ rods expressed PIGCY with only moderate cell-to-cell mosaicism (Fig. 7*B*), and the average expression level of PIGCY evaluated qualitatively (Fig. 7*A*) is similar to the endogenous GCAP1 present in *PIGCY*^*Tg*^ rods, so it generally still remains a fairly minor protein in the outer segment. There is also no detectable aggregation of PIGCY in the inner segment or cell body (Fig. 8) that could conceivably provoke apoptosis *via* massive, unfolded protein/heat shock–like responses. A longer-term characterization of the retina anatomy and function remains to be addressed in future studies, but the initial assessment of the *PIGCY*^*Tg*^ retina (Fig. 10) does not raise major concerns about potential negative effect on survival of photoreceptors for prospective translational studies of PIGCY effects in various animal models.

It also remains to be determined in detailed future electrophysiological studies how the inhibitory effect of PIGCY on RetGC regulation affects the light response in mice harboring RetGC1 mutations that cause overproduction of cGMP. Even complete elimination of the GCAP-stimulated activity drastically changing photoresponse in *GCAPs*^*−/−*^ rods (42, 57) helps *R838S*^*Tg*^ photoreceptors survive (16). We expect that the partial reduction of RetGC activity in the negative Ca^2+^ feedback observed *in vitro* (Figs. 6 and 9) could alter the shape of *PIGCY*^*Tg*^ rod responses. Yet, it is also important that the residual activity of the cyclase in *PIGCY*^*Tg*^ rods still remains stimulated by low Ca^2+^, in contrast to *GCAPs*^*−/−*^ rods (Fig. 9 and ref. (9, 42)). Due to a complete lack of the negative Ca^2+^-feedback on RetGC, light responses in *GCAPs*^*−/−*^ rods saturate at much lower than the normal light intensities (42, 57). Substantial reduction but not complete elimination of the negative Ca^2+^-feedback in *PIGCY*^*Tg*^ retinas suggests that the shape of their rod responses may become somewhat similar to *GCAPs*^*−/*^^*−*^ yet changed less drastically than *GCAPs-*^*−/−*^. The electrophysiological studies to elucidate the potential changes in *PIGCY*^*Tg*^ photoresponse have been initiated.

There are still some other questions that remain to be addressed in future studies related to the inhibition of RetGC1 by PIGCY *in vivo*. We find it somewhat intriguing that PIGCY expression at the level similar to the endogenous GCAP1 suppress RetGC activity in *PIGCY*^*Tg*^ retinas not as strongly as one could expect from its inhibitory activity demonstrated *in vitro*, at higher GCAP1:PIGCY molar ratios (Figure 3, Figure 4, Figure 5). Although not precisely quantified (and also considering some mosaicism of the expression), average PIGCY expression level in *PIGCY*^*Tg*^ rods is definitely not an order of magnitude lower than GCAP1 present in those rods and on average is quite similar to GCAP1 (Fig. 7, *A* and *B*). We do not have a good explanation for this effect at this time, but this may indicate that the native RetGC undergoes some additional, presently unidentified interactions with GCAP1 *versus* PIGCY when their complexes are assembled in a living photoreceptor. GCAPs can also undergo partial dimerization (58), although it remains unclear if this property plays a definitive functional role in regulation of RetGC1 by GCAP1. It is unclear at present if GCAP1 and PIGCY can form mixed dimers. *In vitro* experiments utilizing recombinant GCAP1 and PIGCY (Figure 3, Figure 4, Figure 5), in which PIGCY counteracts the activation by GCAP1 even in the presence of a large molar excess of GCAP1, do not seem to favor a possibility that GCAP1 would reduce the effect of PIGCY by simply “scavenging” it into a less inhibitory complex, but we cannot *a priori* exclude that such or similar interactions may occur *in vivo*. This and other possibilities remain to be subjects of further biochemical studies.

Developing PIGCY as a specific inhibitor of retinal guanylyl cyclase in the outer segment now also opens an opportunity to test its potential ability to rescue photoreceptor degeneration caused by adCORD–linked mutations in *GUCY2D* and/or *GUCA1A* using animal models. Such long-term studies are presently in progress.

## Experimental procedures

### Animals

All experiments involving animals were conducted in accordance with the Public Health Service guidelines and approved by the Drexel University Institutional Animal Care and Use Committees. The wildtype C57BL/6J mouse strain originated from JAX Research/Jackson’s Laboratory. *GCAPs*^*−/−*^ mice, produced by replacing *Guca1a* and *Guca1b* genes adjacent to each other with a single knockout cassette (42), originated from Dr Jeannie Chen laboratory (USC), *GCAP1*^*−/−*^*, GCAP2*^*−/−*^, and *R838S*^*Tg*^ mice were produced as described previously (35, 59, 60). All gene knockout mice were made congenic with the C57BL/6J genetic background by breeding for over 10 generations prior to conducting the experiments. Mice were fed the same diet and were housed in the same temperature- and humidity-controlled environment using 12 h/12h light/dark cycle.

### GCAP1 mutagenesis to produce PIGCY

Mutations were introduced in bovine GCAP1 cDNA by “splicing by overlap extension” technique using PCR reactions catalyzed by high-fidelity Phusion Flash polymerase (ThermoFisher). The resultant DNA fragments were ligated into the NcoI/BamHI sites of pET11d (Novagene/Calbiochem) vector, sequenced by Sanger method, and transformed into expressing cell lines as described previously in detail (22).

### Expression and purification of GCAP and PIGCY

Myristoylated bovine GCAP1 and its mutant variants for the *in vitro* assays were expressed from pET11d vector in a BLR(DE3) *E. coli* strain (both originated from Novagen/Calbiochem) harboring a pBB131 plasmid coding for a yeast N-myristoyl transferase. The proteins were isolated from inclusion bodies by urea extraction, refolded, and subsequently purified using hydrophobic interaction and size-exclusion chromatography, as previously described in detail (27). The purity of recombinant proteins estimated by SDS gel electrophoresis was ≥ 90%.

### Producing PIGCY^Tg^ transgenic mouse model

*PIGCY*^*Tg*^ mice were developed using random DNA insertion approach. PIGCY cDNA containing GCAP1 Kozak motif was assembled in, a previously used for transgenic expressions, a Stratagene pBluescript plasmid backbone, positioned between 4.2-kb mouse rhodopsin promoter and 0.5-kb mouse protamine 1 gene fragment containing polyadenylation signal (16, 21). PIGCY coding cassette containing the promoter, the PIGCY cDNA, and the polyadenylation signal was excised using the *PvuI/XbaI* digestion, purified, and ultimately injected in male pronuclei of fertilized C57BL/6J mouse eggs (service provided by Taconic/Cyagen Biomodels) to develop F_0_ founders. The founders were genotyped by PCR using tail DNA samples and two primers, 5′- TCTGAGCCTGGTCCTCAAGC and 5′- CCTGCGC-ACAATGCGGGTAAA. Three F_0_ founders passed the PIGCY transgene to progeny, and two of them expressed PIGCY in the retina at similar levels. One of these two founders was subsequently outcrossed to C57BL/6J to establish the *PIGCY*^*Tg*^ line.

### Isolation of rod outer segments

ROS fraction for testing PIGCY activity was isolated from wildtype mice using Optiprep density gradient centrifugation as described in detail previously (9). Rhodopsin concentration in ROS fraction or whole retinas was determined using A_500_ absorbance (61). An aliquot of the ROS preparation or retina homogenate was mixed with equal volume of Tris buffered saline containing 2.3% N,N-dimethyl-n-dodecylamine N-oxide and 1 mM hydroxylamine, incubated 5 min in the dark at room temperature, and centrifuged for 5 min at 10,000*g*, 4 °C to remove the insoluble debris, and the absorbance of solubilized rhodopsin at 500 nm in the supernatant was measured before and after complete bleaching by white light. The concentration of rhodopsin was calculated assuming ε∼40,000 M^−1^ cm^−1^ and molecular mass 39 kDa (ca. 1 mg/ml rhodopsin/1 optical unit ΔA_500_).

### RetGC activity assays

Recombinant RetGC activity in HEK293 cells transfected with a human RetGC1 cDNA was measured under ambient illumination using a crude membrane fraction isolated from the transfected cells as previously described in detail (24, 29). The native RetGC activity in mouse retinas was assayed as previously described in detail (9). The retinas for RetGC activity measurements were excised from 1.5- to 2-months-old dark-adapted mice, euthanized under infrared illumination, using a dissecting microscope fitted with infrared goggles. Retinas were frozen in liquid N_2_ and stored at −70 °C prior to their use in the cyclase activity assays, which was also conducted under infrared illumination. RetGC assay mixtures (25 μl) containing HEK293 membranes, retinal homogenates, or ROS membranes in 30 mM MOPS–KOH (pH 7.2), 60 mM KCl, 4 mM NaCl, 1 mM DTT, 2 mM Ca^2+^/Mg^2+^/EGTA buffers, free Mg^2+^ as indicated in the text, 0.3 mM ATP, 4 mM cGMP, 1 mM GTP, and ∼1 μCi of [α–^32^P]GTP, 100 μM zaprinast and dipyridamole, and 10 mM creatine phosphate/0.5 unit of creatine phosphokinase (Sigma-Aldrich/Millipore) were incubated at 30 °C for 40 min in case of HEK293 cell membranes or 12 min in case of retinal preparations, and the reaction was stopped by heat-inactivation at 95 °C for 2 min. The resultant [^32^P]cGMP product was separated by TLC using fluorescently backed polyethyleneimine cellulose plates (Merck) developed in 0.2 M LiCl, cut from the plate, and eluted with 0.5 ml of 2 M LiCl in scintillation vials, and the radioactivity was counted using liquid scintillation. The assay contained ∼0.5 μCi [^3^H]cGMP as the internal standard to ensure the lack of the cGMP product hydrolysis by phosphodiesterases. Ca^2+^/EGTA buffers for measuring Ca^2+^ sensitivity of RetGC were prepared as previously described (62) using Tsien and Pozzan method (63). Data fitting was performed using a Synergy Kaleidagraph 4 software.

### Antibodies

Anti-GCAP1 (RRID: AB_3668998) and anti-RetGC (RRID:AB_2877058) rabbit polyclonal antibodies were characterized previously and validated using transgenic mouse models (9, 59, 60); rabbit polyclonal anti-peripherin 2 antibody was a generous gift from Dr Andrew Goldberg (Oakland University). Secondary peroxidase-conjugated goat anti-rabbit antibody was purchased from Pierce/ThermoFisher, and AlexaFluor488 and AlexaFluor 568 anti-rabbit antibodies for immunofluorescence microscopy were purchased from Invitrogen/ThermoFisher.

### Immunoblotting

The retina homogenates from mice equilibrated by rhodopsin content were subjected to immunoblotting after extraction in AbCam Radio Immuno Protein Assay mixture of detergents supplemented with protease inhibitors cocktail (Millipore/Sigma). The samples mixed with 5:1 volume of 5 × Laemmli SDS sample buffer (Millipore/Sigma) were subjected to electrophoresis in 4 to 20% or 16% gradient polyacrylamide gel (Invitrogen/Thermo Fisher), next to a set of prestained PageRuler Plus molecular mass markers (Pierce/ThermoFisher) using running buffer containing 0.1% SDS and 0.5 mM EGTA. Following the electrophoresis, the proteins were transferred overnight at 50V constant voltage to Immobilon P membrane (Millipore) at 18 °C using Tris-glycine transfer buffer (Fisher Scientific). The membrane was washed with Tris-buffered saline (Fisher Scientific) containing 0.5% Tween-20 (TTBS), blocked by SuperBlock (ThermoFisher) solution in TTBS, and probed by primary antibody for 1 h at room temperature, followed by washing and probing with the secondary antibody. The secondary antibody was removed by washing three times x 15 min in TTBS and twice in Tris-buffered saline. The luminescence signal was developed using peroxidase-conjugated secondary polyclonal anti-rabbit antibody and a Pierce SuperSignal Femto substrate reagent (ThermoScientific). The images were acquired and processed using a Fotodyne Luminous FX imager and ImageJ (National Institutes of Health) software. The profiles of nonsaturated luminescence intensity on the blot were graphed by scanning the gel images using Gel Analyzer of the ImageJ.

### Cell-based PIGCY/RetGC1 colocalization assay

The assay was conducted as originally described in detail in (31) with modifications subsequently described in (22, 24, 53). In brief, HEK293 cells were transfected in LabTeck 4-well cover glass chamber with 1 μg of mOrangeRetGC1 DNA per well using 3 μl/μg DNA of the Promega FuGENE reagent following the protocol recommended by the manufacturer at ∼1:100 M ratio of PIGCY-GFP or GCAP1-GFP coding plasmids *versus* mOrangeRetGC1 coding plasmid or a control plasmid not containing RetGC1 cDNA, ∼1 μg total DNA/well. Confocal images were typically taken after 24 to 32 h of incubation in 5% CO_2_, 37 °C, utilizing an Olympus FV1000 Spectral instrument with the respective 543 nm and 488 nm excitation wavelengths for the red and the green fluorochromes. Images collected in a sequential mode were superimposed on differential interference contrast and processed using Olympus FluoView FV10-ASW software. No changes to the original images were made except for minor gamma correction applied to whole image for more clear presentation in print. When required, the fluorescence was assigned color blind-friendly pseudo colors.

### PIGCY immunofluorescence in the retina

Mice aged ca. 1.5 months were euthanized by lethal injection of ketamine/xylazine and then perfused through the heart, first with PBS, and then with 5% formaldehyde fixative solution in PBS. Enucleated eyes were processed and embedded for cryosectioning as previously described (21). The cryosections were washed three times in PBS containing 0.1 M glycine (pH 7.4), blocked for 1 hour at 30 °C with the same solution containing 5% bovine serum albumin and 0.1% Triton X-100, probed with diluted 1:10,000 anti-GCAP1 antibody (59), incubated overnight at 4 °C and then for 1 h at room temperature, then washed with PBS solution three times for 15 min each, incubated with diluted 1:350 fluorescently labeled goat anti-rabbit secondary antibody and, where indicated, fluorescently labeled PNA (Vector laboratories) diluted 1:200 for 1 h at room temperature, then stained with TO-PRO-3 dye in PBS for 15 min, washed with PBS three times for 10 to 15 min at room temperature, and mounted for the subsequent confocal imaging using an Olympus FV1000 Spectral instrument as described above. Retinas for flat-mount immunofluorescence microscopy were prepared as described previously (21) and probed with anti-GCAP1 antibody (1:10,000) and PNA (1:200). Anti-GCAP1 fluorescence in confocal images was assigned green, PNA fluorescence magenta, and TO-PRO-3 either red or blue colors.

### Optical coherence tomography

Mice were anesthetized using intraperitoneal injection of 20 mg/kg ketamine and 8 mg/kg xylazine (PennVet). The pupils were dilated by applying 1% tropicamide and 2.5% phenylephrine ophthalmic eye drops ∼5 to 10 min before the scan. The A- and then B-scans of the retinas were acquired using an iiScience optical coherence tomography camera calibrated by the manufacturer at 2.47 μm/pixel axial scale and 3.5 μm/pixel lateral scale resolution and averaged from ∼200 frames. The thickness of the ONL layer (97% rod nuclei + 3% cone nuclei, ref. (64)), was measured between the outer plexiform and the external limiting membrane reflective layers (65, 66) from the B-scans across the retina between 400 and 700 μm below the optic nerve in the A-scan.

### Statistics

Statistical significance of the differences was evaluated by unpaired Student’s *t* test assuming unequal variance or ANOVA/Tukey *post hoc* test (alpha 0.01) using a Synergy Kaleidagraph software. Where applicable, normality of data distribution was tested by Kolmogorov–Smirnov test using an on-line calculator https://www.socscistatistics.com/tests/kolmogorov/default.aspx. The pertinent statistical differences are presented in Results.

*GCAP1 structural model* was presented using a Schrödinger PyMol software.

## Data availability

The data referred to in this manuscript are contained within the manuscript. Unprocessed data can be available upon reasonable request from the corresponding author (alexander.dizhoor@drexel.edu).

## Conflict of interests

The authors declare that they have no conflicts of interest with the contents of this article.
